# Design of Space Target Surveillance Constellation Based on Simulation Comparison Method

**DOI:** 10.3390/s25133977

**Published:** 2025-06-26

**Authors:** Qinying Hu, Desheng Liu, Ziwei Dong

**Affiliations:** 1 School of Space Information, Space Engineering University, Beijing 101416, China; huqinying_7@outlook.com (Q.H.); dongziwei5201314@163.com (Z.D.); 2 National Key Laboratory of Space Target Awareness, Beijing 101416, China

**Keywords:** space target surveillance, constellation configuration, simulation comparison method

## Abstract

Aiming at the requirement of space situational awareness on the full-domain and all-time coverage capability of low Earth orbit space targets and focusing on the design of a space-based space target awareness system, a space target awareness constellation design method based on simulation comparison is put forward. On the basis of systematically analyzing the distribution of space targets’ orbits, the low Earth orbit space target surveillance mission mode, key indicators, and constraints of space target surveillance constellations are designed, and three space target surveillance constellation basic configurations are constructed. This paper randomly samples surveillance objects based on the stratified space target orbit distribution and selects a heterogeneous space target surveillance constellation in sun-synchronous morning–twilight orbit that meets the requirements of the surveillance mission model and capability by using simulations and comparisons. The experiments show that the constellation can provide a satisfactory observation arc segment for cataloging and orbiting more than 80% of low Earth orbit targets.

## 1. Introduction

With the continuous advancement of science and technology, the pace of human exploration and utilization of space has accelerated, space has become a key domain of competition and strategic confrontation among nations, and space security has become an important component of national security [1]. Space Target Surveillance (STS) is a technological system integrating tracking, identification, analysis, and early warning functions for artificial objects in space, such as satellites, rocket debris, and space fragments. STS is emerging as a crucial capability for a crucial function for maintaining space order and ensuring the safety of aerospace activities:

- Collision Avoidance: Through precise orbital prediction mechanisms, STS triggers real-time satellite evasion maneuvers to effectively prevent collision accidents.
- Satellite Maintenance Support: By leveraging advanced technologies such as thermal imaging and radio frequency (RF) monitoring, STS conducts health monitoring of high-value aerospace assets like communication satellites and meteorological satellites, providing critical technical support for satellite operation and maintenance.
- Space Sustainability Promotion: By continuously tracking the distribution and movement patterns of space debris, STS provides accurate target lists for space debris cataloging and active removal operations, strongly advancing the sustainable development of the space environment.
- Space Military Security Safeguard: Through dynamic tracking and feature recognition of adversarial spacecraft, STS delivers real-time intelligence support to command centers, serving as a core capability for maintaining space military security.

Establishing an effective and comprehensive space target monitoring system and improving space situational awareness capabilities have become a research hotspot. The space target monitoring system can mainly be divided into the ground-based monitoring system and the space-based monitoring system. The space-based monitoring system refers to detecting space targets (including satellites, rocket bodies, debris, etc.) through space-based platforms equipped with observation devices. Although the space-based monitoring system is currently used less frequently than the ground-based monitoring system, it can overcome the limitations of geographical location and weather conditions [2], provide superior space coverage, and, to some extent, enable full-domain, all-time, and all-weather situational awareness of space targets.

To address the low Earth orbit (LEO) target full-area census, this paper designs a space target surveillance constellation via simulation and comparison, based on sun-synchronous dawn–dusk orbits. It integrates optical visibility and arc segment validity constraints to construct configurations for an optimal solution with fewer satellites, high resource efficiency, and superior performance.

Previous studies have focused on multi-orbital Walker constellations, which fail to meet optical requirements due to constraints. This paper’s innovation lies in a constellation of sun-synchronous dawn–dusk orbits at varying altitudes, where satellites provide optimal optical conditions and enhance resource utilization. Additionally, the study classifies targets by altitude and compares constellation capabilities via three performance indicators, offering a more comprehensive evaluation than prior work. Target classification makes the constellation more targeted, facilitating future optimization. This research meets LEO surveillance needs and references future constellation design.

The rest of this paper is structured as follows: Section 2 introduces the space target surveillance satellites currently in practical application and common constellation design methods; Section 3 conducts an in-depth analysis of the mission requirements for space target surveillance; Section 4 introduces the simulation process and performs a comparative analysis of performance indicators based on the simulation results; and Section 5 provides a brief summary of the full text and proposes follow-up research directions.

## 2. Related Research

Since 1996, the United States has been actively developing a space-based surveillance system, resulting in two development paths: close reconnaissance of space targets and extensive surveillance of space targets. It has launched a series of satellites, represented by the SBSS program [3] and the GSSAP program [4], which significantly improved the U.S. space situational awareness capabilities. China has also launched several important research projects and plans focusing on space debris and has deployed a number of satellites for space object surveillance for low-Earth-orbit targets. Currently, the space object surveillance satellites in practical use mainly operate independently. However, with the increase in the number of targets and the demand for timely completion of missions, independent monitoring by a single satellite no longer meets the needs of space and time coverage. As a result, increasing attention is being paid to the design of space-target surveillance constellations in order to balance the need for space-based surveillance capability with economic benefits.

Satellite constellation design is essentially a multi-objective, multi-constraint optimization problem. There are three main methods of satellite constellation design: geometric analysis, simulation comparison, and optimization algorithm [5]. Geometric analytical methods design constellations by analyzing orbital characteristics and using an analytical approach to target coverage properties. Using computer graphics as a tool, Zhang et al. [6] proposed a strategy for designing three-dimensional space-derived situational awareness constellation configurations. Through a grid search, they identified the optimal constellation configuration that simultaneously achieves high coverage and a low number of satellites. However, this method only considered the visibility relationship between satellites and targets, without accounting for payload visibility constraints. The simulation comparison method involves comparing multiple constellation schemes through simulations and selecting the appropriate constellations based on mission objectives. Taking into account the distribution of debris in LEO orbital debris, Du [7] et al. constructed three Walker constellations of different satellites that used a variety of field of views (FOVs) and concluded that a constellation with parameters of 98.7: 12/4/1 could create and maintain a catalog of 200,000 LEO space debris. Payload visibility and signal-to-noise ratio (SNR) limitations are fully considered in this method, but the satellite utilization rate of this constellation was low due to lighting conditions. The design method based on optimization algorithms refers to the use of modern optimization algorithms for constellation design, which is widely applied in current research. Ye et al. [8] transformed the constellation configuration design problem into multiple independent satellite orbit design problems and optimized it with differential evolution algorithms to maximize the entire constellation’s non-repeating target coverage. However, they did not consider the load balancing issue of each satellite in practical applications. Huo [9] used a sun-synchronous orbit as the observation orbit, based on the Walker constellation, and employed a multi-variant position adaptive genetic algorithm for optimization. The final constellation configuration design scheme obtained a high coverage rate, achieving target location convergence. Current research on space target surveillance constellations, on the one hand, lacks consideration of payload constraints in practical applications and an insufficient understanding of the complexity of space target surveillance constellation configuration problems. On the other hand, it is limited to the Walker constellation, which, due to optical visibility constraints, results in low observation efficiency and significant resource waste for constellations designed under this strategy.

## 3. Analysis of the Requirements of Space Target Surveillance

At the beginning of the space target surveillance constellation design, the space-based surveillance mission should first be clarified, and the requirements of the space target surveillance mission should be analyzed. This serves as input for designing satellite orbits and constellation configurations. Three main aspects are considered: the characteristics of the surveilled targets, the objectives of the surveillance mission, and the objective constraints.

### 3.1. Surveillance Target Characteristics

Space targets can be classified according to their orbital characteristics, which are typically described by the orbital six elements:

- Semi-major axis (a): The length of the major axis of the orbital ellipse.
- Eccentricity (e): A parameter characterizing the deviation of the orbit from a circular shape.
- Inclination (i): The angle between the orbital plane and the Earth’s equatorial plane.
- Right ascension of the ascending node (Ω): The angle between the Earth’s vernal equinox direction and the ascending node (the point where the satellite crosses the equatorial plane from south to north).
- Argument of perigee (ω): The angle between the ascending node and the perigee (the point closest to Earth in the orbit).
- True anomaly (ν): The angle between the perigee and the satellite’s current position, measured at the Earth’s center.

According to statistics from the US website www.Celestrak.com (accessed on 1 April 2025), there are 11,213 satellites currently in orbit. Table 1 shows the distribution of space objects.

Low Earth orbit (LEO) targets account for approximately 92.90% of all targets, with the vast majority of man-made objects, including Earth observation satellites, communication satellites, and space stations, operating in low Earth orbit. On one hand, a large number of operational satellites are widely distributed in LEO, and these satellites move at a faster speed compared to those in other orbits, which results in relatively shorter observable arcs for space-based surveillance satellites. On the other hand, due to satellite disintegration and collision events, a significant amount of space debris is generated and remains in orbit for extended periods, further increasing the potential for collisions with low Earth orbit targets. Additionally, some satellites have orbit-changing and close-approach phenomena. Therefore, low Earth orbit targets are the primary focus of space target surveillance constellations.

**Figure 1:**
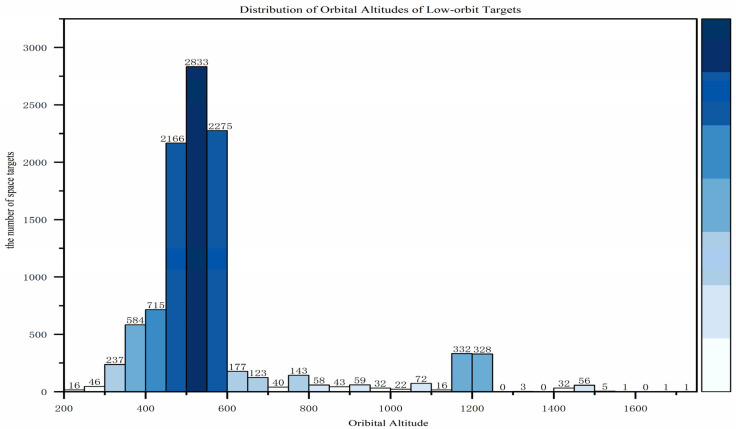
Distribution of orbital altitudes of low-orbit targets.

**Figure 2:**
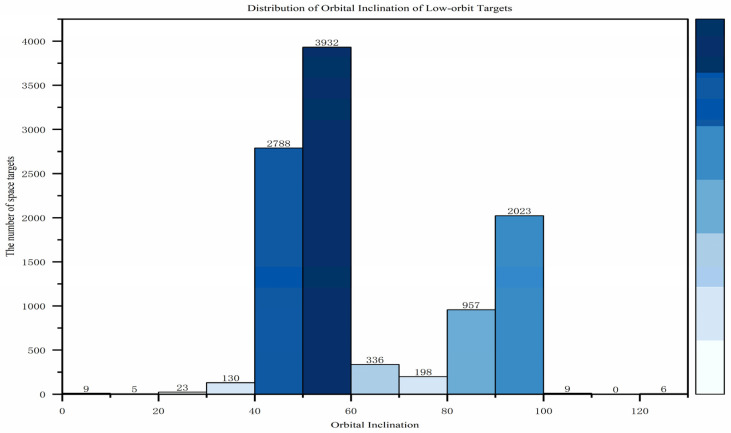
Distribution of orbital inclinations of low-orbit targets.

**Figure 3:**
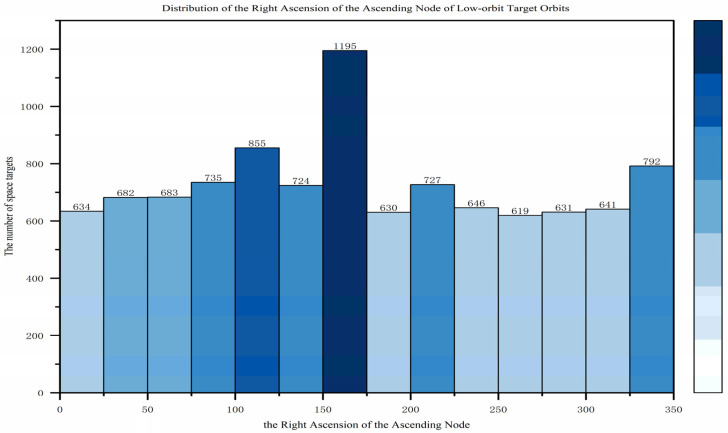
Distribution of the right ascension of the ascending node of low-orbit target orbits.

This section primarily analyzes the characteristics of low Earth orbit (LEO) target distribution from three aspects: orbital altitude, orbital inclination, and the right ascension of the ascending node. In terms of orbital altitude, as shown in Figure 1, with the large-scale launch and orbital insertion of SpaceX’s Starlink satellites, 85.1% of low Earth orbit targets have an average orbital altitude below 600 km, peaking around 550 km. Regarding orbital inclination, as shown in Figure 2, the distribution is concentrated in two ranges: [40,60] and [90,100], with the former being suitable for most satellites covering densely populated regions in the mid-to-low latitudes, while the latter is primarily used for sun-synchronous orbits. As for the right ascension of the ascending node, as shown in Figure 3, compared to the orbital inclination and altitude, the distribution of the ascending node’s right ascension for low Earth orbit targets is relatively uniform, making full use of the limited orbital resources.

Due to the enormous number of low Earth orbit targets, if all low Earth orbit targets are used as samples for simulating the space target monitoring constellation, the computational efficiency would be extremely low. Stratification is an effective sampling method that can effectively suppress the uneven distribution of parameters in the sample population, thereby reducing differences between units and improving sampling accuracy through independent random sampling. Therefore, this paper uses stratified random sampling to obtain samples, which are then taken as the mission objects of the space target surveillance constellation. Based on the analysis of the characteristics of low Earth orbit targets distribution mentioned earlier, the orbital inclination is used as the stratification criterion, as shown in Table 2, which is mainly divided into the following four layers.

Based on the distribution characteristics of low Earth orbit targets, this paper determines the total sample size as 1012 using relative error limits for estimation accuracy and allocates sample sizes for each layer by proportional allocation, which are 16, 653, 145, and 198, respectively. Then, the target orbits for the samples are determined by random sampling within each layer.

### 3.2. Surveillance Task Requirements

The main purpose of the low-orbit space target surveillance system is to perceive the attitude–orbit status and target characteristics of space targets in real time, analyze and judge the targets’ movement trend and evolutionary direction, provide support for state monitoring, collision early warning, and health management of space targets, and serve the management of space traffic to ensure the safety of space assets. From the perspective of mission objectives and capability requirements, space target surveillance can be categorized into three types of tasks, as described below.

The first is the full-area census. This type of task is mainly used to obtain orbital characteristic data of space targets to support orbit cataloging, anomaly analysis, and trend research and judgment. The task capability requirements mainly include indicators such as spatial coverage, target revisit interval, and observable rate.

The second is the detailed investigation. This type of task is mainly used to observe the target characteristics of space targets (such as geometry, radiation characteristics, payload, attitude, etc.) and support the characterization and health diagnosis of space targets. The mission capability requirements mainly include indicators such as imaging resolution and attitude measurement accuracy.

The third is dynamic tracking. This type of mission is mainly used to carry out gaze tracking of specific space targets, provide real-time position information of the targets, and offer real-time target indication for the measurement, transportation, and control of space targets, rendezvous, and docking, as well as the capture and cleanup of space debris. The mission capability needs mainly include tracking duration, tracking accuracy, and other indicators.

In this paper, we focus on the first type of mission as the traction requirement, and take meeting the observation data requirement of orbit cataloging as the design basis, focusing on the design indexes such as revisit interval, observable rate, and coverage ratio, among which the coverage ratio is an indicator used to evaluate the constellation’s performance in monitoring multiple space targets, while the others are used to evaluate the constellation’s performance in monitoring a single space target.

- Revisit Interval

Due to the characteristics of low Earth orbit (LEO) targets, such as low orbital altitude and high velocity, and the potential for orbital maneuvering, there are certain time requirements for adjacent observation arcs in space surveillance missions targeting LEO objects. The revisit interval $T_{revisit}$ refers to the time gap between two consecutive observation arcs of a space target by the surveillance constellation, as shown in Formula (1). Among them, $t_{s}(i+1)$ represents the starting time of the (i + 1)-th observation arc, and $t_{e}(i)$ represents the ending time of the *i*-th observation arc.

(1) $$T_{revisit}=t_{s}(i+1)-t_{e}(i)$$

The shorter the revisit interval, the more continuous the information obtained by the monitoring constellation, such as target position and velocity, leading to smaller errors in target orbit prediction. This can effectively improve the accuracy and reliability of target tracking and orbit determination.

- Observability Rate

The observability rate $P_{observability}$ refers to the ratio of the cumulative arc length of all effective observation segments of a surveillance constellation on a spatial target over one orbital period to the orbital period itself, as shown in Formula (2). Among them, $l_{arc}(i)$ refers to the length of the *i*-th observation arc segment of the spatial target, and its calculation formula is shown in Equation (3). $N_{arc}$ represents the total number of observation arc segments for the spatial target and $T_{regression}$ refers to the orbital period of the spatial target.

(2) $$P_{observability}=\frac{\sum_{i=1}^{N_{arc}}l_{arc}(i)}{T_{regression}}$$

(3) $$l_{arc}(i)=t_{e}(i)-t_{s}(i)$$

The higher the observability rate, the richer the observational data obtained by the surveillance constellation during one orbital period of the target. This leads to a more comprehensive understanding of the target’s state information and enhances the effectiveness of space target monitoring. Furthermore, when tracking and monitoring a specific space target, a higher observability rate means greater flexibility in mission planning, allowing for more precise scheduling of observation times and resources. This enables the formulation of a more reasonable mission plan and helps to achieve an optimal monitoring strategy.

- Coverage Ratio

The coverage ratio $P_{\mathrm{cov}erage}$ refers to the ratio of the number of space targets observable by the monitoring constellation during the simulation period to the number of space target samples in the monitoring task. Its calculation formula is shown in Equation (4). Among them, $N_{observability}$ refers to the number of space targets observable by the surveillance constellation during the simulation time period, while $N_{sample}$ is the total number of space target samples.

(4) $$P_{\mathrm{cov}erage}=\frac{N_{observability}}{N_{sample}}$$

The higher the coverage ratio, the more satellite targets the surveillance constellation can effectively observe, leading to a broader surveillance range and stronger target capture capabilities; thus, the completeness of the surveillance task increases.

### 3.3. Analysis of Constraints

#### 3.3.1. Optical Visibility Constraints

**Figure 4:**
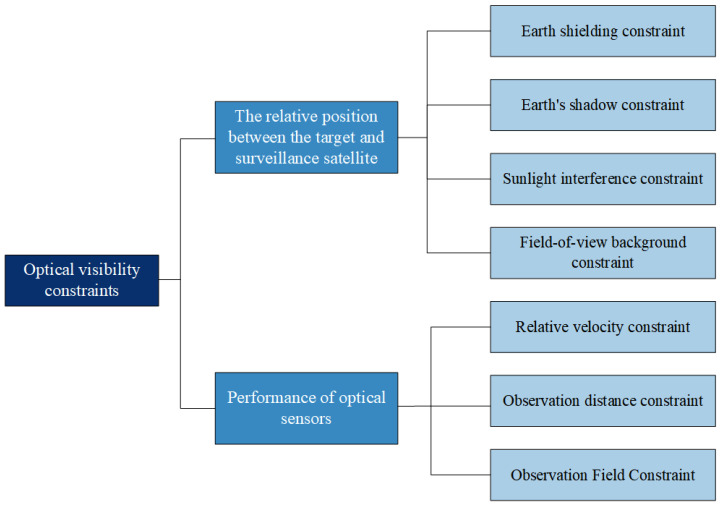
Optical visibility constraints.

Visible light observation satellites rely on the reflection of visible light by the target to achieve observation. They have advantages such as stable operation and being passive, but they are limited by constraints related to optical visibility. This paper mainly considers the following constraints, as shown in Figure 4.

- Earth shielding constraint

The Earth shielding constraint refers to a situation where the target is capable of reflecting sunlight, but the reflected light is blocked by the Earth and its atmosphere during propagation, causing the target to become invisible.

- Earth’s shadow constraint

The Earth’s shadow constraint refers to the situation where a target is located in the Earth’s shadow zone, making it impossible to reflect sunlight and thus invisible. The shadow zone refers to the area formed on the opposite side of the Earth, where sunlight is blocked and the Earth casts a shadow.

- Sunlight interference constraint

The sunlight interference constraint refers to the situation where the target is observed against the backdrop of the sun, and the sun’s brightness causes severe interference with the optical sensor, making normal imaging impossible.

- Field-of-view background constraint

Surveillance satellites often use the position of stars to determine target angle information. Field-of-view background constraints refer to situations where, when the satellite’s orbit is higher than the target, the Earth may become the field-of-view background and thus interfere with the measurement of target angle information.

- Relative velocity constraint

Due to the constraints of the optical sensor’s performance, the relative speed between the target and the surveillance satellite cannot exceed the maximum relative speed at which the sensor can identify the target.

- Observation distance constraint

The observation distance constraint refers to the fact that, due to the limited observation range of optical sensors, the distance between the target and the surveillance satellite must be less than the observation limit distance.

- Observation Field Constraint

The observation field constraint refers to the limitation of the target being within the surveillance satellite’s observation range due to the field of view restrictions of optical sensors.

#### 3.3.2. Arc Segment Validity Constraint

Space target orbit determination can mainly be divided into initial orbit determination and precise orbit determination. Initial orbit determination refers to the process of estimating the initial orbit using short arc observation data and employing simple, fast, and simplified dynamic orbit determination methods. Precise orbit determination refers to the process of improving the dynamic model’s inaccurate parameters using sufficient observational data due to the inaccuracies in the initial orbital elements and perturbation equations, so that the orbital parameters are accurately determined [10].

The traditional initial orbit determination methods can essentially be categorized into two types: Laplace and Gauss methods. These are typically suitable for arc lengths longer than one minute. However, for very short arcs, such as those lasting only a few dozen seconds or even shorter, these two algorithms and their derivatives often fail to converge to a solution. To address this issue, Liu [11] improved the Laplace method and studied the requirements for arc length in the initial orbit determination of space targets. When the success criterion for orbit determination is that the semi-major axis error is less than 100 km, the required initial orbit arc segment lengths are shown in Table 3. Zhang [12] et al. applied neural network algorithms to enhance the accuracy of traditional initial orbit calculation methods, solving data for observation arcs as short as 21 s. Approximately 98% of the targets achieved final calculation accuracies better than 20 km. Lei [13] proposed a geometry-based initial orbit association method that does not require error covariance. For low Earth orbit satellites monitoring low-orbit targets, with an average arc length of 23 s, the association accuracy was 84.0% when the time interval between the two initial orbits was less than 3 days.

The main function of a space target surveillance constellation is to provide arc segment data for the detection and maintenance of space object orbit determination and cataloging. Space-based surveillance systems, especially LEO monitoring constellations, are limited by the relative velocity effect when observing LEO targets, often resulting in short observation arc segments. To ensure the provision of effective arc segment data, there are validity constraints on the length of observation arcs and the time intervals between adjacent arcs. Specifically, the observation arc length for space targets by surveillance satellites $l_{arc}$ cannot be shorter than the minimum observation arc length required by the mission $l_{arc}^{\min}$, as shown in Equation (5), and the time interval between adjacent observation arcs $T_{revisit}$ cannot exceed the maximum revisit interval required by the mission $T_{revisit}^{\max}$, as shown in Equation (6).

(5) $$l_{arc}\geq l_{arc}^{\min}$$

(6) $$T_{revisit}\leq T_{revisit}^{\max}$$

## 4. Simulation Experiments and Result Analysis

### 4.1. Constellation Schemes

Currently, most space-based target surveillance satellites in operation adopt sun-synchronous orbits, particularly the sun-synchronous dawn–dusk orbits, where the descending node local time is 6:00 or 18:00, such as the Canadian satellite ‘Sapphire’ and the first satellite of the U.S. SBSS program, Block-10, which use sun-synchronous dawn–dusk orbits at altitudes of 786 km and 630 km, respectively. On one hand, optical sensors are currently a feasible payload option for space-based target surveillance constellations, as sun-synchronous dawn–dusk orbits provide and maintain relatively optimal lighting conditions. On the other hand, the average movement of sun-synchronous orbit satellites is as high as 12–15 orbits per day, offering frequent revisit opportunities for targets within their coverage area. Therefore, this paper designs a constellation configuration based on the sun-synchronous dawn–dusk orbit.

**Figure 5:**
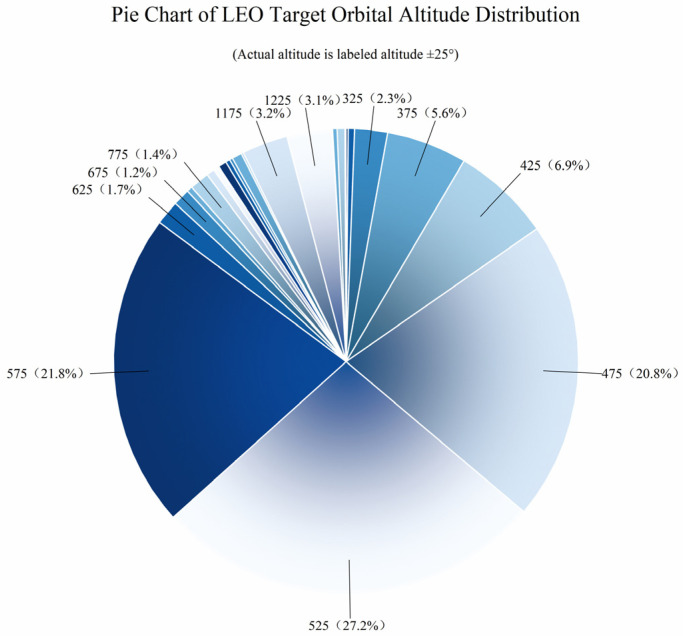
Pie Chart of LEO target orbital altitude distribution.

As analyzed in Section 3.1 on the characteristics of low Earth orbit target distribution and as shown in Figure 5, low Earth orbit targets are concentrated within the range of [450,600] km. A constellation of multiple satellites with an orbital altitude of 550 km is selected as the primary monitoring constellation to mainly observe lower-altitude satellites. At the same time, considering that some low Earth satellites are distributed at higher altitudes, several satellites with orbital altitudes of 700 km and 1600 km are selected to assist in observing higher-altitude satellites from different angles.

Therefore, this paper designs three different constellations, consisting of either a single Walker constellation or a combination of multiple Walker constellations. All satellites adopt a sun-synchronous dawn–dusk orbit and carry the same onboard payload. The specific constellation configurations and the attributes of the onboard payload are detailed in Table 4 and Table 5.

**Figure 6:**
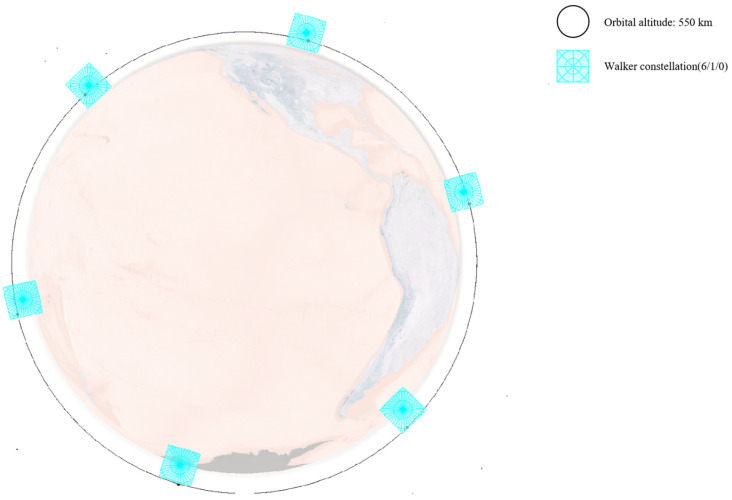
Configuration schematics of CON.1.

**Figure 7:**
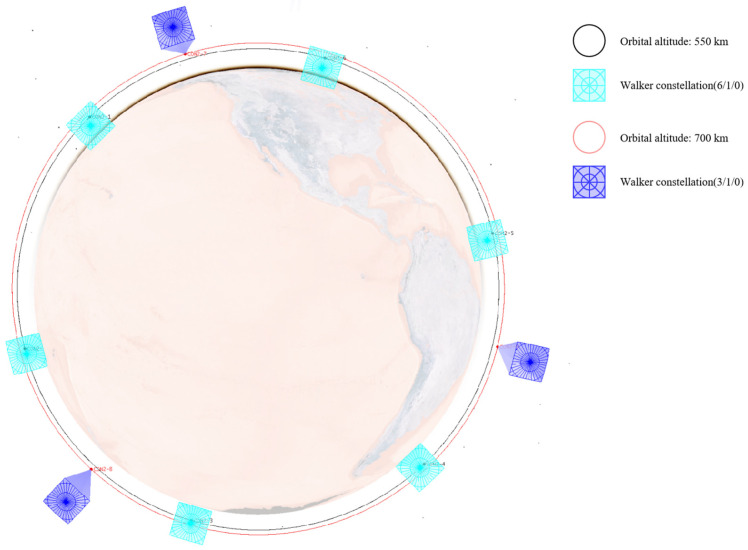
Configuration schematics of CON.2.

**Figure 8:**
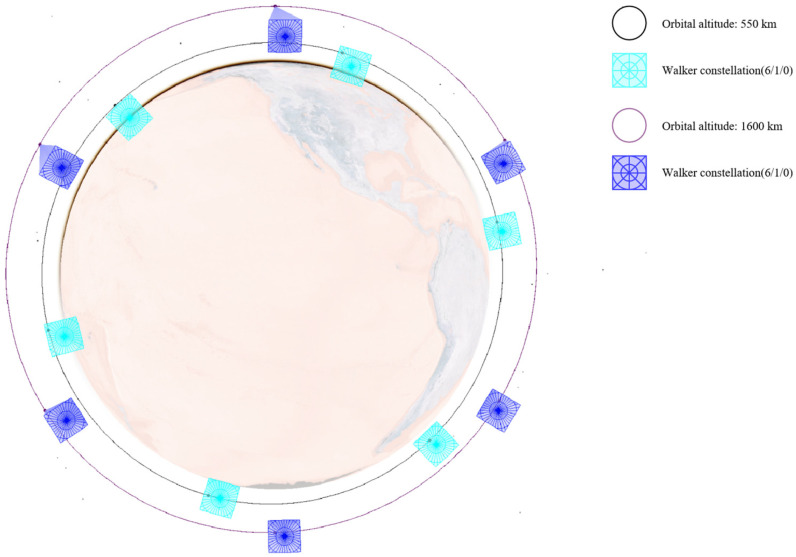
Configuration schematics of CON.3.

Their configuration schematics are shown in Figure 6, Figure 7 and Figure 8. Different colors represent different orbital altitudes.

### 4.2. Simulation Process

The simulation period for this experiment was from 2 April 2025 to 4 April 2025, lasting a total of 48 h. A random stratified sampling method was used to select 1012 TLE catalog targets from 10,417 low Earth orbit targets as the simulation targets. The simulation process is as follows:

(1) Define the orbital parameters for each simulated constellation and the sensor specifications for the satellites. Generate a reference satellite with fixed altitude in a sun-synchronous dawn–dusk orbit as the seed model, then use it to initialize a Walker constellation. By configuring Walker constellation parameters (T/P/F), the software systematically replicates the seed satellite to form a multi-satellite constellation.

For multiple heterogeneous Walker constellations, the aforementioned procedure is replicated for each configuration. Specifically, a reference satellite at a specific orbital altitude is generated as a seed satellite, and Walker constellation parameters (T/P/F) are configured to generate a single-altitude constellation. These single-altitude constellations are then integrated into a unified, multi-altitude surveillance architecture.

As specified in Table 5, we configure the telescope parameters, including aperture, field of view (FOV), and resolution. The primary optical axis of the telescope is directed in the sunlight direction and perpendicular to the orbital plane to optimize illumination conditions. From this baseline:

- In the CON.1 constellation, the optical axis of each satellite is deflected by 10° toward the Earth’s center.
- For the CON.2 constellation, the satellite at 550 km altitude features a 10° optical axis deflection toward the Earth’s center, whereas the satellite at 700 km altitude has a 10° deflection away from the Earth’s center.
- In the CON.3 constellation, the 550 km altitude satellite exhibits a 10° optical axis deflection toward the Earth’s center, and the 1600 km altitude satellite has a 15° deflection in the same direction.

**Figure 9:**
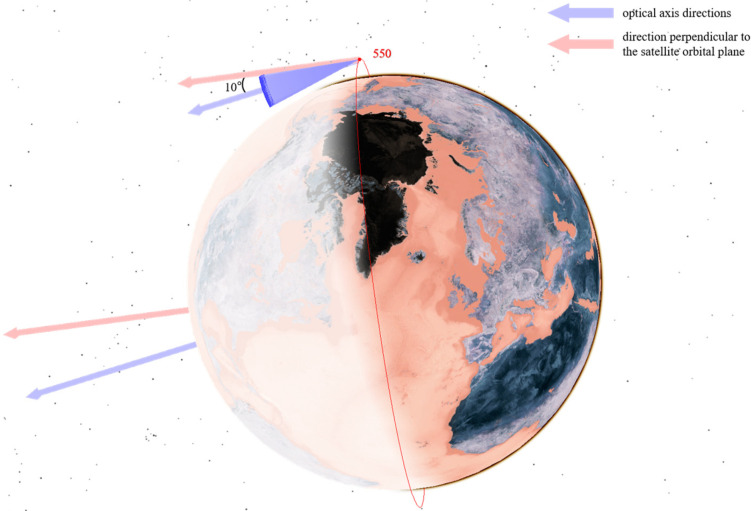
Optical axis pointing of satellites at 550 km.

**Figure 10:**
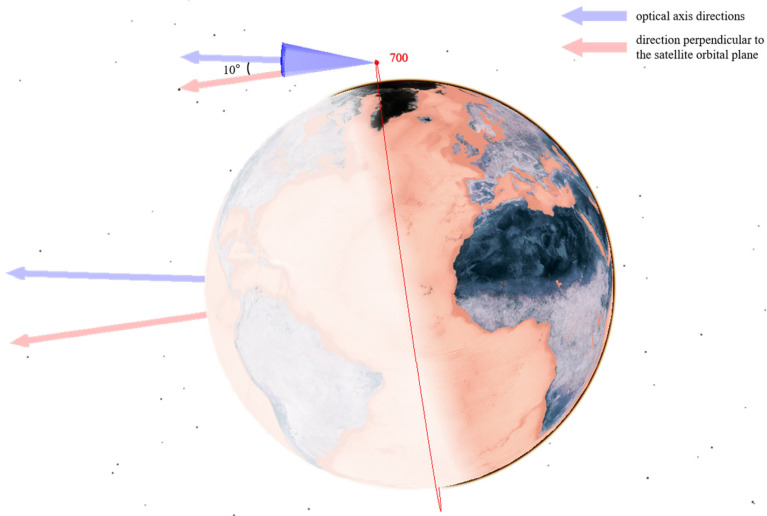
Optical axis pointing of satellites at 700 km.

**Figure 11:**
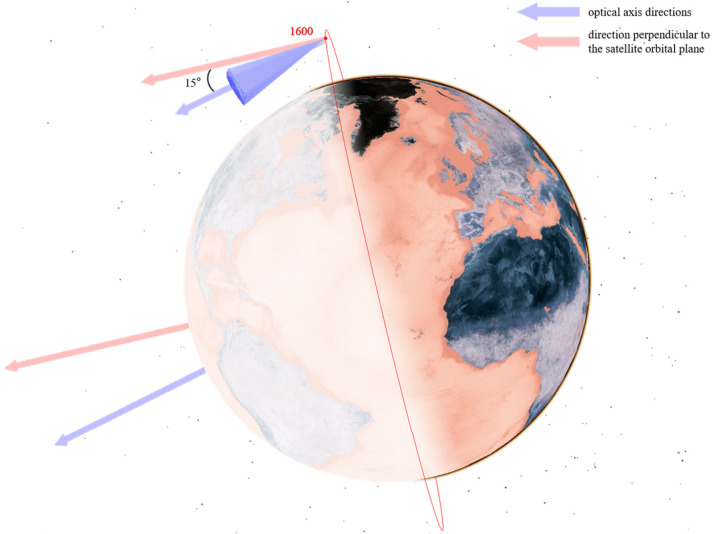
Optical axis pointing of satellites at 1600 km.

The optical axis orientations for satellites at different orbital altitudes are illustrated in Figure 9, Figure 10 and Figure 11.

(2) Predict the positions of the simulated target and the satellite. Use the two-line elements (TLE) of both the simulated target and the satellite in conjunction with the SGP4 orbit prediction model to obtain their positions during the simulation period at 10-s intervals.

(3) Generate visible arcs with optical constraints. Apply optical visibility constraints (Earth occlusion, Earth shadow, field-of-view background, relative velocity, observation distance, and observation field) to the telescope and simulation targets. Obtain the visible arc segments that the positional relationship between the two satisfies the constraints and output the calculation result.

(4) Analyze simulation results. Analyze the simulation results based on performance indicators such as the revisit interval, observability rate, and coverage ratio, and objectively evaluate and compare the three constellations.

### 4.3. Performance Analysis

Due to the short simulation period, this paper determines whether a target is covered based on whether it meets the arc length constraints. These criteria are based on Liu’s research (see Table 3 for details): during the simulation period, a target is deemed covered if either (1) a single surveillance satellite has a continuous observation arc length of over 400 s for the target or (2) for the same target, two or more surveillance satellites each have a continuous observation arc length of over 40 s. If neither condition is satisfied, the target is deemed uncovered.

To further analyze the targeting of the three constellations, based on the distribution characteristics of LEO targets, the LEO target samples are divided into three categories according to the orbital altitude: below 450 km, between 450 km and 600 km, and above 600 km. Table 6 details the performance of the three constellations for different target categories in terms of the average revisit interval, average observability rate, and coverage ratio. This section analyzes the applicability of the three constellations based on these three performance indicators.

#### 4.3.1. Average Revisit Intervals

**Figure 12:**
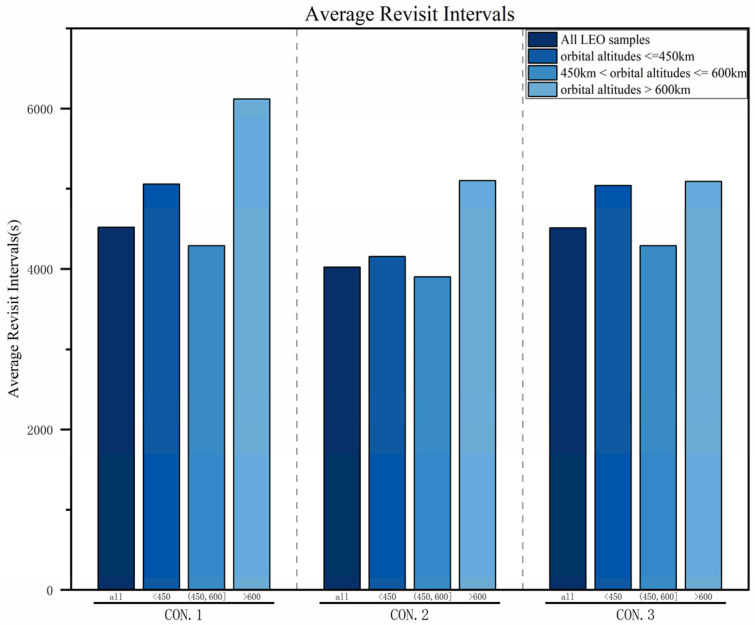
Comparison of average revisit intervals.

As shown in Figure 12, for all LEO target samples, the average revisit interval of CON.2 is significantly shorter than that of CON.1 and CON.3. Compared with CON.1, CON.2 adds three satellites at a 700 km altitude, which not only increases the number of repeated observations for lower-orbit targets but also raises the probability of observing higher-orbit satellites. Therefore, for LEO target samples at different orbital altitudes, the average revisit interval of CON.2 is shorter than that of CON.1. Compared with CON.1, CON.3 uses six satellites at a 1600 km altitude to improve the observation efficiency of higher-orbit satellites, significantly reducing the average revisit interval for LEO target samples with altitudes > 600 km. However, due to the longer orbital period, satellites at a 1600 km altitude have a relatively low probability of revisiting LEO target samples at altitudes < 600 km, resulting in the average revisit interval not decreasing significantly. Overall, the average revisit intervals of all three constellations for LEO space targets at any orbital altitude are less than 2 h, which essentially meets the requirements for tracking and monitoring arc segments.

#### 4.3.2. Average Observability Rates

**Figure 13:**
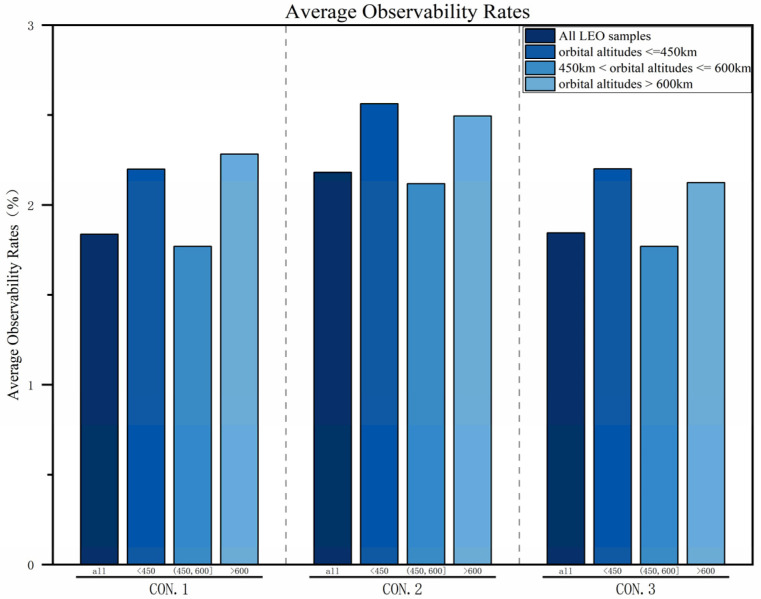
Comparison of average observability rates.

As shown in Figure 13, the proportion of observation arc segments relative to the orbital period of space objects is relatively low for all three constellations. The main reason is that the relative speed between LEO satellites and space targets is high, making each observation arc segment short, and the number of revisits within a single orbital period is limited. Overall, compared with CON.1 and CON.3, CON.2 has a higher average observability rate for LEO space object samples at different orbital altitudes. On one hand, CON.2 adds three satellites at a 700 km altitude based on CON.1, increasing the probability of observing space objects. On the other hand, the three satellites at 700 km in CON.2 have shorter orbital periods compared to the satellites in CON.3 at a 1600 km altitude. For target samples with orbital altitudes > 600 km, CON.2 has a higher probability of repeating observations within a single orbital period, thus improving the observability of the target.

#### 4.3.3. Coverage Ratios

**Figure 14:**
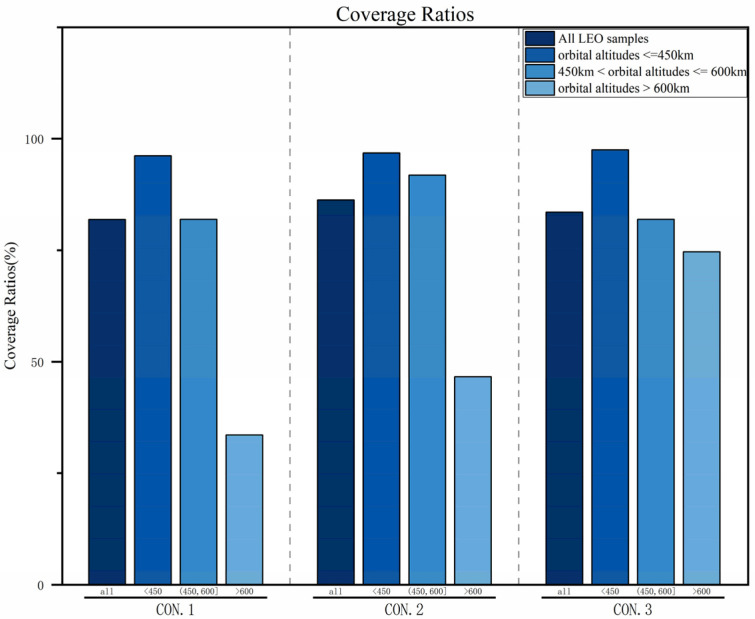
Comparison of coverage ratios.

As shown in Figure 14, the coverage ratios of the three constellations for different target types are compared. Based on the LEO target distribution discussed in Section 3.1, the constellations are designed with orbital altitudes of 550 km (where targets are relatively clustered), 700 km (a distribution gap), and 1600 km (the maximum target altitude found). To better observe the targets, the satellites’ optical axes have been appropriately adjusted. Compared with CON.1, CON.2 significantly improves the coverage ratio for target samples at orbital altitudes > 450 km. This improvement mainly stems from the fact that monitoring satellites at a 550 km altitude are largely limited to targets not exceeding this orbital height, resulting in lower tracking and observation probabilities—and thus lower coverage—for targets at other altitudes. Compared with both CON.1 and CON.2, CON.3 greatly increases the coverage ratio for target samples > 600 km, reaching 74.66%, which represents improvements of 41.1% and 28.08%, respectively.

Through comprehensive comparison, if the primary focus of the monitoring task is on targets with orbital altitudes < 600 km (mid–low-orbit LEO targets), CON.2 is the preferred choice. This space object surveillance constellation offers a shorter average revisit interval, a higher average observability rate, and covers more than 90% of such targets. If the focus is on targets with orbital altitudes > 600 km (mid–high-orbit LEO targets), CON.3 should be prioritized. Although this constellation is limited by a longer orbital period and does not exhibit an extremely high average observability rate during the simulation period, it provides a higher coverage ratio for targets > 600 km and maintains a shorter average revisit interval, thus meeting the monitoring task requirements.

**Figure 15:**
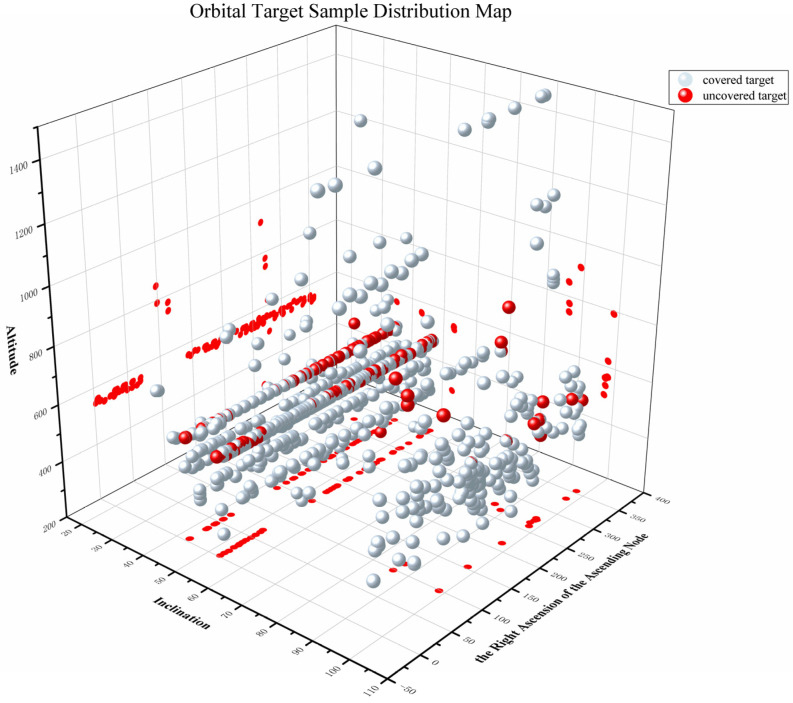
Distribution of space target samples (the X-, Y-, and Z-axes represent inclination, the right ascension of the ascending node, and altitude of target samples, respectively).

Figure 15 shows the coverage of LEO targets by CON.3, where red solid circles represent uncovered targets. It is evident that the orbits of the uncovered targets are mostly below 600 km, predominantly concentrated around 500 km. Their orbital inclinations mostly range between 40° and 55°. Considering the coverage characteristics of the three constellations, as well as economic benefits, constellation size, and coverage ratio, future work could involve forming a heterogeneous constellation with satellites at 550 km, 700 km, and 1600 km altitudes, carefully designing the number of satellites at each altitude. Alternatively, the overall performance indicators of the surveillance constellation could be improved by adjusting the payload orientation or optimizing payload performance.

## 5. Conclusions

This paper summarizes the current development and application of space target surveillance constellations, starting from the distribution of LEO space targets. A heterogeneous space monitoring constellation based on sun-synchronous dawn–dusk orbits is proposed by integrating mission requirements, analyzing constellation constraints, and defining performance indicators with mathematical expressions. The simulation focuses on 550-km-altitude satellites as the main body, with additional 700-km-altitude and 1600-km-altitude satellites for auxiliary observation. By analyzing the simulation results, the performance indicators of the three constellations for different target categories are compared, and the suitable constellations for targets at different orbital altitudes are summarized. As the basic constellation of this paper, CON.1 has better optical observation conditions and significantly improved constellation resource utilization compared with constellations proposed in other studies. It can achieve basic coverage of targets below 450 km. As improved versions of CON.1, CON.2 and CON.3 enhance the coverage of targets at other orbital altitudes. For example, CON.2 greatly improves the coverage of targets with altitudes between 450 km and 600 km, enabling better observation of mid-low-orbit LEO targets. In contrast, CON.3 significantly improves the coverage of targets with orbital altitudes higher than 1600 km, making it suitable for observing mid–high-orbit LEO targets. Additionally, uncovered targets are concentrated near the 500 km altitude, suggesting future improvements through constellation configuration optimization or payload performance enhancement.

Additionally, performance analysis shows that the three constellations have low single-target observability across orbital altitudes. The main challenge is poor observation arc continuity, caused by high relative velocities between the constellation and targets. We propose two improvements:

- Optimize payloads for a wider FOV and a longer observation range.
- Integrate space-based and ground-based systems for multi-source surveillance, leveraging their combined advantages.

This study provides a methodological reference for the design of space-based space target surveillance constellations. Future research can further integrate on-orbit resource scheduling and task planning to enhance the overall effectiveness of the constellation.

## Figures and Tables

**Table 1:** Distribution of Space Objects.

Target Type	Grouping Criteria	Percentage of All Targets(%)
LEO	a < 8378 km	92.90
MEO	8378 km < a < 27,378 km	1.14
HEO	27,378 km < a < 42,078 km	0.70
GEO	a > 42,078 km and i < 15°	4.98
IGSO	a > 42,078 km and i > 15°	0.28

The ‘a’ represents the semi-major axis of the target orbit, and ‘i’ represents the inclination of the target orbit.

**Table 2:** Targets’ orbit stratification.

Layer Serial Number (h)	Stratification Criterion	Total Quantity of the hth Layer (N_h_)	Percentage of LEO Targets(%)
1	i < 40°	167	1.61
2	40° <= i < 60	6720	64.51
3	60° <= i < 90°	1491	14.31
4	90° <= i	2039	19.57

The ‘i’ represents the inclination of the target orbit.

**Table 3:** Requirements for the length of the initial orbit determination arc segment of space targets by low-orbit surveillance satellites.

Target Type	Minimum Observation Arc Length (Single Satellite)(s)	Minimum Observation Arc Length (Double Satellites)(s)
LEO	400	40
GEO	1700	450

**Table 4:** Configuration of low Earth orbit surveillance constellations.

Constellation	Orbital Inclination (°)	Orbital Altitude (km)	T	P	F	Orbital Type
CON.1	97.6	550	6	1	0	Sun-synchronous dawn-dusk orbit
CON.2	97.6	550	6	1	0	Sun-synchronous dawn-dusk orbit
	98.2	700	3	1	0	Sun-synchronous dawn-dusk orbit
CON.3	97.6	550	6	1	0	Sun-synchronous dawn-dusk orbit
	102.51	1600	6	1	0	Sun-synchronous dawn-dusk orbit

**Table 5:** Optical payload attributes.

Payload	Distance (km)	Imaging Resolution	Aperture (mm)	Lateral Relative Velocity (km/s)	Field of View (°)	Angular Resolution (″)
CCD/CMOS	300	4096 × 4096	120	3	9 × 9	8.8

**Table 6:** Comparison of performance indicators.

Constellation	Simulation Targets	Average Revisit Interval (s)	Average Observability Rate (%)	Coverage Ratio (%)
CON.1	All LEO target samples	4522.37	1.84	81.90
	orbital altitude < 450 km	5057.14	2.20	96.18
	orbital altitude between 450 and 600 km	4290.40	1.77	81.95
	orbital altitude > 600 km	6117.76	2.28	33.56
CON.2	All LEO target samples	4022.99	2.18	86.27
	orbital altitude < 450 km	4157.13	2.56	96.82
	orbital altitude between 450 and 600 km	3901.40	2.12	91.82
	orbital altitude > 600 km	5100.86	2.49	46.58
CON.3	All LEO target samples	4512.63	1.84	83.50
	orbital altitude < 450 km	5040.86	2.20	97.45
	orbital altitude between 450 and 600 km	4290.40	1.77	81.95
	orbital altitude > 600 km	5091.60	2.12	74.66

## Data Availability

The data presented in this study are available on request from the corresponding author. The data are not publicly available due to private reasons.
